# Revised contraindications for the use of non-medical WB-electromyostimulation. Evidence-based German consensus recommendations

**DOI:** 10.3389/fspor.2024.1371723

**Published:** 2024-04-16

**Authors:** S. von Stengel, M. Fröhlich, O. Ludwig, C. Eifler, J. Berger, H. Kleinöder, F. Micke, B. Wegener, C. Zinner, F. C. Mooren, M. Teschler, A. Filipovic, S. Müller, K. England, J. Vatter, S. Authenrieth, M. Kohl, W. Kemmler

**Affiliations:** ^1^ Institute of Radiology, University Hospital Erlangen, Erlangen, Germany; ^2^ Department of Sports Science, Rheinland-Pfälzische Technische Universität Kaiserslautern-Landau, Kaiserslautern, Germany; ^3^ German University for Prevention and Health Management, Saarbrücken, Germany; ^4^ Institute of Training Science and Sport Informatics, German Sport University Cologne, Cologne, Germany; ^5^ Musculoskeletal University Center, Ludwig-Maximilian-University of Munich, Munich, Germany; ^6^ Hessian College of Police and Administration, Wiesbaden, Germany; ^7^ Department of Rehabilitation Sciences, Faculty of Health, University of Witten/Herdecke, Witten, Germany; ^8^ Soccer Club Paderborn 07, Paderborn, Germany; ^9^ Glucker Kolleg, Frankfurt, Germany; ^10^ Bundeswehr Medical Academy Munich, Munich, Germany; ^11^ PT Lounge Cologne, Cologne, Germany; ^12^ EMS-Performance, Kornwestheim, Germany; ^13^ Department of Medical and Life Sciences, University of Furtwangen, Schwenningen, Germany

**Keywords:** whole-body electromyostimulation, contraindications, diabetes mellitus, cancer, neurologic diseases, arteriosclerosis

## Abstract

Whole-body electromyostimulation has proven to be a highly effective alternative to conventional resistance-type exercise training. However, due to adverse effects in the past, very extensive contraindications have been put in place for the commercial, non-medical WB-EMS market. Considering recent positive innovations e.g., federal regulation, mandatory trainer education, revised guidelines, and new scientific studies on WB-EMS application, we believe that a careful revision of the very restrictive contraindications on WB-EMS is needed. This applies all the more because many cohorts with limited options for conventional exercise have so far been excluded. During a first meeting of an evidence-based consensus process, stakeholders from various backgrounds (e.g., research, education, application) set the priorities for revising the contraindications. We decided to focus on four categories of absolute contraindications: “Arteriosclerosis, arterial circulation disorders”, “Diabetes mellitus” (DM), “Tumor and cancer” (TC), “Neurologic diseases, neuronal disorders, epilepsy”. Based on scientific studies, quality criteria, safety aspects and benefit/risk assessment of the category, DM and TC were moved to the relative contraindication catalogue, while arteriosclerosis/arterial circulation disorders and neurologic diseases/neuronal disorders/epilepsy were still considered as absolute contraindications. While missing evidence suggests maintaining the status of neurologic diseases/neuronal disorders as an absolute contraindication, the risk/benefit-ratio does not support the application of WB-EMS in people with arteriosclerosis/arterial circulation diseases. Despite these very cautious modifications, countries with less restrictive structures for non-medical WB-EMS should consider our approach critically before implementing the present revisions. Considering further the largely increased amount of WB-EMS trials we advice regular updates of the present contraindication list.

## 1 Introduction

Whole-body electromyostimulation (WB-EMS) is a recognized training technology that focuses mainly on functional, body composition and health-related outcomes in nonathletic adults (1). Due to its joint friendliness and time efficiency, WB-EMS can be considered as an attractive option for users otherwise unable or unmotivated to exercise conventionally. However, the unique feature of WB-EMS being able to stimulate large muscle areas simultaneously but with dedicated in excess supra-maximum impulse intensity for each region carries the inherent risk of over-straining and adverse effects at least after inadequate WB-EMS application (2, 3). In this context, “the recommended contraindications for the use of non-medical WB-Electromyostimulation” was released by a German expert group in 2019 (4), in order to prevent WB-EMS application in vulnerable cohorts. The limited regulation of WB-EMS, non-mandatory instructor education and evidence gaps on conditions and diseases considered particularly critically for WB-EMS application in essence led to a very restrictive list of absolute contraindications being advised (Table 1). In the last few years however, several positive innovations have fundamentally impacted the commercial non-medical German WB-EMS market. This includes in particular a federal ordinance^1^ regulating WB-EMS application (5) and mandatory trainer education (6), but also to updated international consensus recommendations for safe and effective whole-body electromyostimulation (7). Considering further that new studies have provided evidence for safe WB-EMS application in cohorts with conditions and diseases absolutely contraindicated to WB-EMS so far, we feel that a revision of the present contraindications is called for so as to carefully open WB-EMS application to people with otherwise limited options and/or motivation for conventional exercise. This might particularly refer to people with arteriosclerosis/arterial circulation disorders, diabetes mellitus, tumor and cancer, neurologic diseases, all absolutely contraindicated to commercial, non-medical WB-EMS. Thus, the aim of the present article is to critically revise the present list of contraindications for WB-EMS application and finally release an updated list of contraindications for WB-EMS based on an evidence driven consensus approach.

**Table 1:** Absolute contraindications for WB-EMS (2016). Contraindications printed in bold and italic were subjected to the revision process.

• Acute diseases, bacterial infections, inflammatory processes
• Recently performed operations in stimulation areas
• ***Arteriosclerosis, arterial circulation disorders***
• Stents and bypasses active for less than 6 months
• Untreated hypertension
• ***Diabetes mellitus***
• Pregnancy
• Electric implants, cardiac pacemakers
• Heart arrhythmia
• ***Tumor and cancer***
• Severe bleeding disorders, tendency of bleeding (hemophilia)^2^
• ***Neurologic diseases, neuronal disorders, epilepsy***
• Abdominal wall and inguinal hernia
• Acute influence of alcohol, drugs and intoxicants

## 2 Material and methods

The present revision of the German contraindications on WB-EMS (4) was coordinated by the Institute of Radiology, University Hospital Erlangen, Germany. For the consensus-based decision-making processes on WB-EMS contraindications, we invited German stakeholders of varying backgrounds. Apart from the leading (German) research groups on WB-EMS, we contacted all accredited educational institutions responsible for the education of WB-EMS trainers. Additionally, two selected WB-EMS studios with long experience of commercial, non-medical WB-EMS were included in the consensus process.

During a kick-off meeting in March 2023, our consortium decided to focus on the revision of absolute contraindications for WB-EMS. The list of present absolute contraindications was discussed and the priorities for revisions were fixed (Table 1). Due to their high prevalence, socioeconomic impact, limited options for intensive conventional exercise and their persistent character (in contrast to the acute or rapidly reversible contraindications listed in Table 1), we decided to focus on four categories of absolute contraindications: “Arteriosclerosis, arterial circulation disorders”, “Diabetes mellitus”, “Tumor and cancer”, “Neurologic diseases, neuronal disorders, epilepsy” (Table 1)^2^,^3^.

### 2.1 Systematic review of the literature

The generation of evidence for WB-EMS application based on a systematic review and evidence map of the literature in the area of WB-EMS intervention studies described in detail in a previous study (1). Briefly, study reports from five electronic databases (Medline [PubMed], The Cochrane Central Register of Controlled Trials [CENTRAL], Cumulative Index to Nursing & Allied Health [CINAHL via Ebsco Host], SPORTDiscus (via Ebsco Host) and The Physiotherapy Evidence Database), two study registers [Clinical trial.gov and the WHO's International Clinical Trials Registry Platform (ICTRP)] published up to 6th March 2023 were searched without language restrictions. To identify additional study reports, we searched Google Scholar manually on the same date as the medical databases.

#### 2.1.1 Eligibility criteria

**Figure 1:**
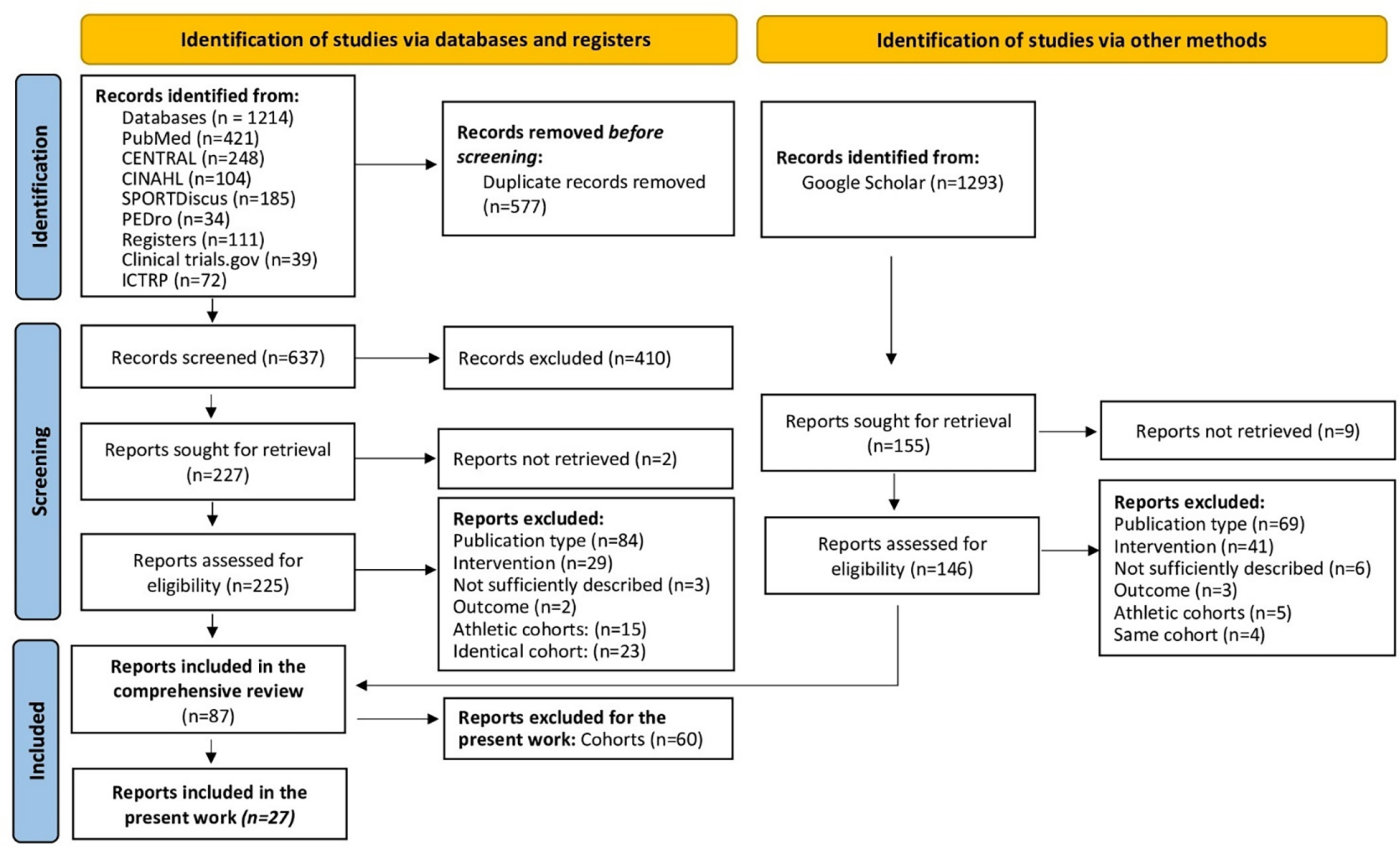
Flow diagram (9) of the comprehensive search (1) adjusted for the present research issue.

Eligibility criteria structured according to PICOS (8) were: (*Population*) Studies with sedentary to non-athletic adult cohorts on average 45 years and older. Studies with athletes or sport students were excluded (Figure 1).

 Of importance, for the present work we extended our eligibility criteria and focus to cohorts with “Arteriosclerosis, arterial circulation disorders”, “Diabetes mellitus”, “Tumor and cancer”, “Neurologic diseases, neuronal disorders, epilepsy” and closely related conditions (e.g., the Metabolic Syndrome) (Figure 1) using the comprehensive search process (1) as a basis. (*Intervention*) Studies that applied Whole-Body Electromyostimulation [WB-EMS (10)] or other kinds of electromyostimulation that can stimulate large muscle areas simultaneously^4^. Studies that applied local EMS or focus on single muscle groups were not considered. (*Comparators*) Type or even presence of a control group was not considered as an eligibility criterion. (*Outcomes*) With few exceptions (e.g., “Anti-G-Straining”) the search process (1) included eligible studies independently of the outcomes addressed (Figure 1). Special emphasis was placed on adverse effects of WB-EMS application. We defined “adverse event” as any untoward medical occurrence, unintended disease or injury. Muscular soreness, discomfort with the stimulation, or increased CK values without clinical relevance were not considered adverse effects. (*Study design*) All types of longitudinal studies with an interventional study designs (11), i.e., randomized or non-randomized clinical trials and intervention studies with or without control groups, were included. Only peer reviewed research was considered.

#### 2.1.2 Selection process

Titles, abstracts and full texts were independently screened by two reviewers according to the pre-specified eligibility criteria listed above. Diseases and conditions were classified according to the International Statistical Classification of Diseases and Related Health Problems (ICD-10 GM). We also recorded whether the outcome was defined as the primary/main study outcomes or as secondary/subordinate study endpoints by the authors. To properly address this issue we carefully checked the article but also the study registration and databases where applicable. Disagreements were solved by discussion or with the help of a third reviewer. Reasons for excluding ineligible studies were recorded. In the case of missing data or doubtful information, authors were contacted for a maximum of three times within a 6-week period. We applied the latest version of the DeepL pro translator (Cologne, Germany) for the translation of articles not in English or German language.

Studies identified by the search process (Figure 1) were screened and categorized for study, cohort, participant, exercise and stimulation characteristics (Table 2).

**Table 2:** Selected characteristics of the included studies.

Authors	Study-design	Total sample size (*n*)	Gender	Age (years)	Training-status	Diseases	EMS-system	Intervention length (months)	Sessions/week (*n*)	Session length (min)	Impulse freque-ncy (Hz)	Drop-out (%)	Adherence (%)	Adverse effects	Methodological quality
Bellia et al. (12)	RCT	25	m + w	49 ± 7	Moderate	MetS	WB-EMS	6	2	20	15 or 85	23	90	No	Low
di Cagno et al. (13)	RCT	24	m + w	72 ± 6	Untrained	Neuro	WB-EMS	3	2	20	7 or 85	0	100	No	High
Fritzsche et al. (14)	IS no CG	15	m + w	27–73	Untrained	CAD	WB-EMS	6	2	20	80	0	n.g.	No	Low
Hamada et al. (15)	NRCT	43	m + w	20–69	Untrained	Ca	B-SES	1	7	20	20	12	71	No	Low
Homma et al. (16)	RCT	27	m + w	79 ± 6	Untrained	NIDDM, CAD	B-SES	3	3	40	20	29	100	No	Mod
Houdijk et al. (17)	NRCT	75	m + w	45–75	Untrained	NIDDM	WB-EMS	4	2	20	85	0	95	No	Low
Imaoka et al. (18)	RCT	49	m + w	64 ± 7	Untrained	NIDDM	B-SES	0.5	5	20	20	27	n.g.	No	Mod
Kataoka et al. (19)	RCT-	16	m + w	83 ± 6	Untrained	Stroke	B-SES	3	3	20	4	25	n.g.	No	Mod
Kemmler et al. (20)	RCT	28	m	69 ± 3	Untrained	MetS	WB-EMS	3.5	1.5	30	85	7	78	No	Mod
Lukashevich (21)	RCT	52	w	45–65	Untrained	Stroke	WB-EMS	0.66	4	20	25,000	n.g.	n.g.	No	Low
Matsumoto et al. (22)	IS no CG	4	m + w	66 ±** **6	Untrained	NIDDM	B-SES	1	5	20	20	n.g.	n.g.	No	Low
Matsuo et al. (23)	NRCT	90	m + w	77 ±** **11	Untrained	CAD, NIDDM	B-SES	0.5	5	20	20	6	94	No	Low
Mori et al. (24)	NRCT	14	m	65 ±** **13	Untrained	Neuro	B-SES	1.5	2	30	20	n.g.	n.g.	n.g.	Low
Nakamura et al. (25)	RCT	94	m + w	76 ±** **12	Untrained	CAD	B-SES	0.5	7	20	20	55	100	n.g.	Low
Nakamura et al. (26)	RCT	68	m + w	68 ±** **15	Untrained	CAD	B-SES	0.5	7	20	20	17	100	n.g.	Mod
Ochiai (27)	NRCT	6	m + w	60–90	Untrained	CAD	B-SES	1.1	7	20	20	0	n.g.	No	Low
Reljic et al. (28)	RCT	103	m + w	≥18	Moderate	MetS	WB-EMS	3	2	20	85	23	93	No	Mod
Richter (29)	NRCT	75	m + w	≥18	Untrained	CA	WB-EMS	3	2	20	85	19	88	No	Low
Schink et al. (30)	NRCT	131	m + w	≥18	Untrained	CA	WB-EMS	3	2	20	85	40	87	No	Low
Schink et al. (31)	NRCT	31	m + w	≥18	Untrained	CA	WB-EMS	3	2	20	85	59	77	No	Low
Schwappacher et al. (32)	NRCT	18	m	≥18	Untrained	CA	WB-EMS	3	2	20	85	n.g.	88	No	Low
Schwappacher et al. (32)	NRCT	12	m + w	≥18	Untrained	CA	WB-EMS	3	2	20	85	n.g.	85	No	Low
Schwappacher et al. (33)	NRCT	12	m + w	>18	Untrained	CA	WB-EMS	3	2	20	85	n.g.	79	No	Low
Suzuki et al. (34)	RCT	29	m + w	65 ± 7	n.g.	CA, NIDDM	B-SES	2	3	20	20	13	98	No	Low
Suzuki et al. (35)	IS no CG	12	m + w	66 ± 10	Untrained	NIDDM	B-SES	3	3	30	20	0	n.g.	No	Low
Tanaka et al. (36)	RCT	39	m + w	>75	Untrained	CAD	B-SES	0.30	5	35	20	25	86	No	Mod
Tsurumi et al. (37)	RCT	22	m + w	74 ± 5	Untrained	NIDDM	B-SES	3	3	30	4	27	n.g.	n.g.	Mod
van Buuren et al. (38)	NRCT	59	m + w	61 ± 13	Untrained	CAD	WB-EMS	2.5	2	20	80	0	100	No	Low
van Buuren et al. (39)	IS no CG	15	m + w	62 ± 3	Untrained	NIDDM	WB-EMS	2.5	2	20	80	0	100	No	Low

B-SES, belt electrode-skeletal muscle electrical stimulation; CA, cancer and tumor; CAD, coronary artery disease, arterial circulation disorders; CG, control group; IS, intervention study; m, men; MetS, metabolic syndrome; mod, moderate; n.g., not given/reported; Neuro, neuronal disease, neuronal disorders, epilepsy; NIDDM, non-insulin dependent diabetes mellitus; NRCT, non-randomized controlled trial; RCT, randomized controlled trial; w, women; methodological quality: studies with >7 score points were classified as high, 5–7 score points moderate (mod) and <5 score points as low methodological quality studies respectively (40).

Methodological quality was rated applying the Physiotherapy Evidence Database (PEDro) Scale Risk of Bias Tool (41), specifically dedicated to physiotherapy and/or exercise studies. Studies with >7 score points were classified as high, 5–7 score points moderate and <5 score points as low methodological quality studies respectively (40) (Table 2). In parallel to the approach listed above, in case of missing data or doubtful information, the authors were contacted for a maximum of three times within a 6-week period.

**Figure 2:**
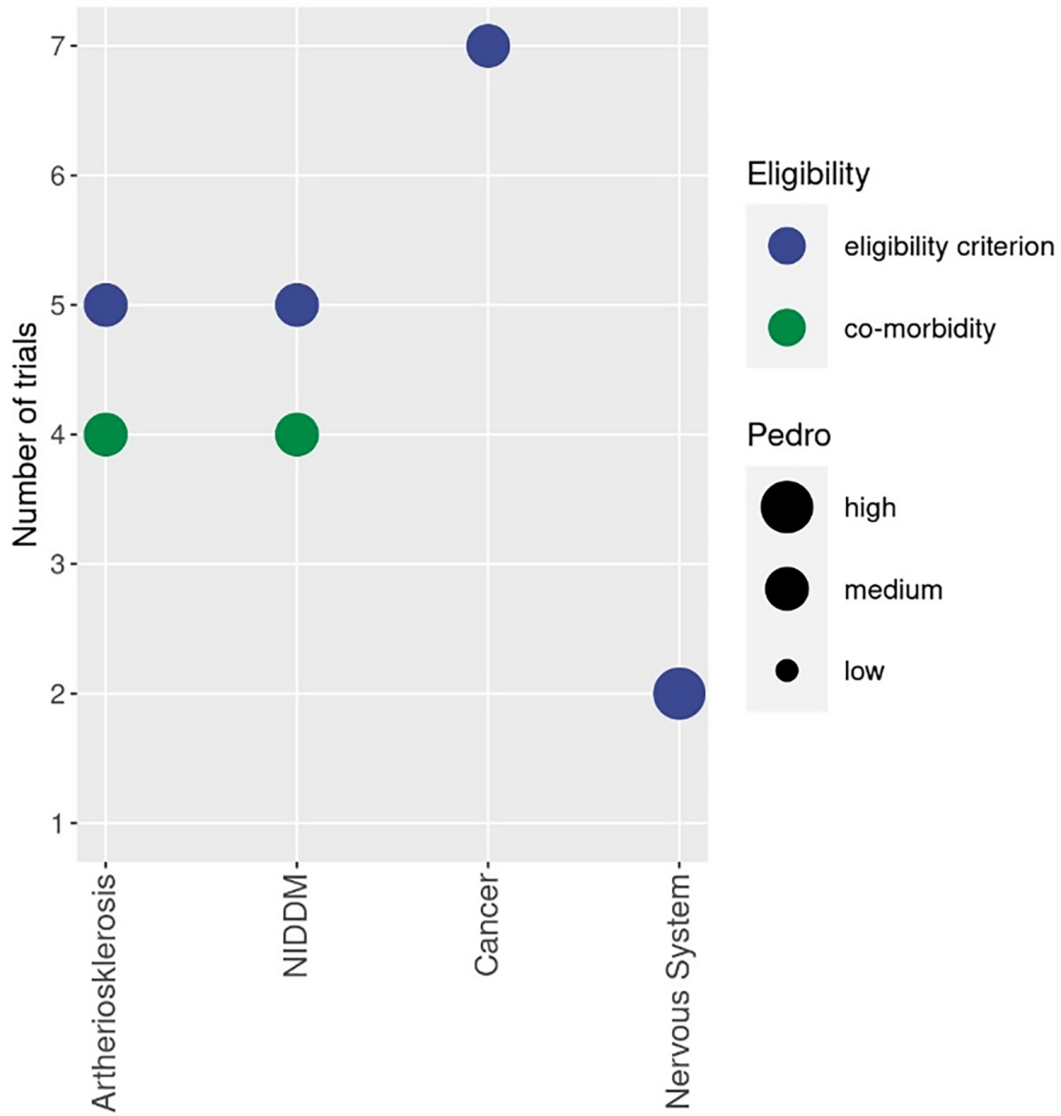
Bubble chart of cohorts with diseases related to the absolute contraindication addressed by WB-EMS studies. Different colours indicate whether the health status of the cohort was applied as a criterion for inclusion (blue) or reported as a simple comorbidity (green). The size of the bubble indicates the methodologic quality according to PEDro (41).

To provide a quick overview, bubble charts with four dimensions were created with the *x*-axis listing the correspondent contraindication and with the *y*-axis presents the number of studies that focus on the corresponding cohort. The shading of the bubble represents whether the health status of the cohort was applied as a criterion for inclusion or reported as a simple co-morbidity (Figure 2).

Finally, the size of the bubble indicates the methodologic quality according to PEDro (41). The biggest size indicates at least one study of high methodologic quality [i.e., PEDro Score ≥8 score points (40)] in the category. The lowest size of the bubble chart represents at least one study of low methodologic quality.

Based on the final analysis, the consortium carefully discussed absolute contraindications prioritized for revision (Table 1). The decision of the consortium was based on the number of studies that addressed the corresponding cohort, quality criteria of the trial and safety aspects with specific regard for adverse effects related to the WB-EMS intervention (Figure 2). The decisive factor was finally the benefit/risk assessment of the category. It was agreed that the recommendation must be made in full consensus and agreement within the consortium.

## 3 Results

Table 2 provided a rough overview of study, cohort, participant, exercise and stimulationcharacteristics of the 27 reports (Figure 1) included in the present work. For a more detailed overview the reader is kindly refered to the comprehensive publication of Beier et al. (1).

Figure 2 displays cohorts, addressed by WB-EMS in the area of absolute contraindication prioritized for revision.

### 3.1 Arteriosclerosis, arterial circulation disorders

Cohorts with atherosclerosis, arterial circulation disorders and related diseases were addressed by several studies (Figure 2). One non-controlled cohort study of 10 weeks (14) and a further non-randomized clinical trial of 4 months (38) included solely participants with chronic heart failure (14, 38). Matsuo et al. (23) and Tanaka et al. (36) selected acute heart failure as an eligibility criterion and applied 10 and 14 days of B-SES during hospitalization in their moderate quality studies. In parallel, about 50% of the critically ill patients of Nakamura et al. (25, 26) and 70% of the hemodialysis patients included in the moderate quality RCT of Homma et al. (16) reported heart failure, cardiopulmonary arrest or had a history of ischemic heart disease (16). The same studies reported that about half of their patients suffered from stroke or displayed a history of cerebrovascular events/disease. Stroke patients <6 months after the stroke event were exclusively addressed by the 3-week RCT of Lukashevich et al. (21). In parallel, about 90% of the bedridden older participants of the RCT of Kataoka et al. (19) suffered from cerebral infarction, cerebral or subarachnoid hemorrhage or hypoxic ischemic encephalopathy. Peripheral arterial diseases/severe ischemia of the lower limbs were an eligibility criterion in two studies (22, 27). Two of the studies (25, 26) with critically ill patients failed to report unintended side effects, none of the studies reported adverse effects related to the intervention. Further, the low methodologic quality of most studies that addressed atherosclerosis/arterial circulation disorders linked diseases as an eligibility criterion for inclusion unfortunately dilutes evidence for applying WB-EMS in conditions related to atherosclerosis and arterial circulation and linked diseases.

### 3.2 Diabetes mellitus

Five randomized and non-randomized trials or intervention studies without CG applied WB-EMS for two to four months in cohorts with non-insulin dependent Diabetes Mellitus (NIDDM) (17, 18, 35, 37, 39)^5^. Two of the studies included hospitalized cohorts with end-stage diabetes kidney disease (37) or diabetic ulcers undergoing minor amputation (18). Additionally, four other trials did not focus on, but included a large proportion of participants with NIDDM (16, 22, 23, 34). Of importance, a further three moderate to high quality RCTs (12, 20, 28) focused on cohorts with the Metabolic Syndrome (MetS) applying WB-EMS for 3–6 months. Unfortunately, one study (37) on NIDDM and the MetS failed to report adverse effects. In summary however, evidence for EMS application in NIDDM can be considered moderate-high. Additionally, three low-moderate quality RCTs that applied MetS as a criterion for inclusion (12, 20, 28) and did not observe adverse effects might increase evidence for WB-EMS application in people with cardiometabolic diseases.

### 3.3 Tumor and cancer

In summary, six studies with seven study groups (15, 29–33) addressed cohorts with malignant neoplasms. In particular, the research group of Zopf et al. (29–33) focused on this issue applying WB-EMS for 12 weeks each. So far, the authors have published data on their ongoing advanced cancer project (30) with subgroup analyses on hematological malignancies (31), gastro-intestinal (29), pancreatic (33), prostate (32) and colorectal cancer (32). According to the authors, results for other tumor entities will be published in the newest future. Hamada et al. (15) focused on patients in the early stage of allogeneic stem cell transplant predominately in people with acute leukemia applying WB-EMS for four post-transplantation weeks^6^. Another study did not focus on, but included cancer patients (34). Of importance, none of the studies reported adverse effects during the intervention. Evidence for WB-EMS application in cancer patients provided by the studies and subgroup-analysis can be considered moderate.

### 3.4 Neurologic diseases, neuronal disorders, epilepsy

Unfortunately, only a few studies focused on cohorts with diseases of the nervous system (13, 24). The high-quality RCT of di Cagno et al. (13) focused on stage 1 (mild) to 3 (moderate) Parkinson's disease in 50–80 years old patients for 12-week. The NRCT of Mori et al. (24) addressed Huntington patients during dialysis with WB-EMS-application for 6 weeks^7^. While di Cagno et al. (13) observed no adverse effects; unfortunately Mori et al. (24) did not report unintended effects of WB-EMS application.

### 3.5 Summary of adverse effects

Four (24–26, 36) of the 27 included studies did not report adverse effects and did not respond to our corresponding queries. Two of these studies addressed the domain of “arteriosclerosis, arterial circulation disorders” (25, 26), one study focused on the domain of “Diabetes Mellitus” (37) and one study addressed “Neurologic diseases, neuronal disorders, epilepsy” (24). All of the studies applied B-SES in a hospital setting with critically ill patients (25, 26), end stage diabetic disease (37) or Huntington patients during hemodialysis (24).

### 3.6 Summary of the consensus process

After a discussion covering (a) the present regulatory framework of WB-EMS in Germany, (b) scientific results on absolute contraindications prioritized by our group and (c) potential harm and negative side effects that could arise if the conditions were to occur, our consortium unanimously decided to move two absolute contraindications, “Diabetes Mellitus” (Type I and II) and “tumor/cancer” to the area of relative contraindications. In particular, due to evidence gaps and severe consequences of adverse effects, we decided to maintain the status of “arteriosclerosis, arterial circulation disorders” and “Neurologic diseases, neuronal disorders, epilepsy” as absolute contraindications (Table 3).

**Table 3:** Revised list of absolute and relative German contraindications for WB-EMS (2024).

Absolute contraindications
• Acute diseases, bacterial infections, inflammatory processes
• Recently performed operations in stimulation areas
• Arteriosclerosis, arterial circulation disorders
• Stents and bypasses active for less than 6 months
• Untreated hypertension
• Pregnancy
• Electric implants, cardiac pacemakers
• Heart arrhythmia
• Severe bleeding disorders, tendency of bleeding (hemophilia)
• Neurologic diseases, neuronal disorders, epilepsy
• Abdominal wall and inguinal hernia
• Acute influence of alcohol, drugs and intoxicants
Relative contraindications
• Diabetes mellitus (Type I and II)
• Tumor and cancer
• Acute back pain without diagnosis
• Acute neuralgia, herniated discs
• Implants older than 6 months
• Diseases of the internal organs particularly kidney diseases
• Cardiovascular diseases
• Movement kinetosis
• Greater fluid retention, oedema
• Open skin injuries, wounds, eczema, burns (in the vicinity of electrodes)
• Corresponding medication for conditions mentioned above

In this context, we define “relative contraindications” as contraindications for which WB-EMS training may only be applied after physician's approval and only with special expertise, licensed education or an adequate medical qualification according to the mandatory NiSV ordinance.

## 4 Discussion

In the present work, our consensus group undertook a very cautious revision of the WB-EMS contraindication list. Finally, only two absolute contraindications, Diabetes Mellitus and tumor/cancer were shifted to the relative contraindication catalogue. Of note, there was an intense discussion about whether cancer/tumor should be completely removed from the contraindications catalog. However due to an ongoing disagreement, the consortium choose the more cautious option. In contrast, arteriosclerosis, arterial circulation disorders and Neurologic diseases, neuronal disorders, epilepsy, were still considered as absolute contraindication. Particularly for the latter cohort a release would have been very welcome considering the low amount of training options for several neurologic limitations and diseases. Nevertheless, we think the rationale for our decision is clear: While missing evidence suggests maintaining the status of Neurologic diseases/neuronal disorders as an absolute contraindication and awaiting further research, in contrast more than a few publications focus on diseases and consequences related to arteriosclerosis/arterial circulation disorders. Nonetheless, considering the severe consequences of adverse effects potentially induced by WB-EMS, the risk/benefit-ratio does not support the use of WB-EMS in people with arteriosclerosis/arterial circulation diseases.

Reviewing other absolute contraindications excluded during round one of the consensus process, i.e., “acute diseases, bacterial infections, inflammatory processes”, “recently performed operations in stimulation areas”, “stents and bypasses active for less than 6 months”, “untreated hypertension”, “pregnancy”, “abdominal wall and inguinal hernia”, “acute influence of alcohol, drugs and intoxicants” should be considered as acute and/or “reversible” contraindications. The latter refer to “untreated hypertension” and in particular “abdominal wall and inguinal hernia” which should receive mandatory medical treatment completely independent of WB-EMS application. Due to severe consequences in case of adverse effects, “electric implants, cardiac pacemakers”, “heart arrhythmia” and “severe bleeding disorders” were also not subjected to the revision. One may argue that our approach was too cautious and there is no or little reason for excluding some cardiovascular and Neurologic diseases from WB-EMS application. We partially agree; however, the present list of contraindication focuses on the use of non-medical whole-body-electromyostimulation. Considering the fast dissemination of medical WB-EMS^8^ in Germany, we think that people with the few remaining absolute contraindications and limited options for other exercises will be able to exercise in this particularly safe setting.

We do not revise the list of relative contraindications so as to retain the physician as the gatekeeper of the process. In this context, one may criticize that most physicians might be unable to estimate the risk and benefits of WB-EMS well enough to release a WB-EMS application. Here, we do not agree. Considering the commercial application since 2007 with thousands of studios, millions of clients and hundreds of publications (1), most physicians are well aware of WB-EMS. We further feel that the physician's willingness to approve WB-EMS application is significantly supported by the reliable framework of German federal directives (5), mandatory trainer education (6) and (hopefully) the non-mandatory guidelines on safe WB-EMS publication (7, 45). In this context, we would like to explicitly point out that our consortium does not endorse any non-physically supervised WB-EMS application (7, 45). This is even more the case for people with limitations, disabilities and diseases who particularly benefit from close supervision and guidance through well-educated trainers.

We would like to draw the reader's attention to a few special features of our approach. Firstly, we also included studies that applied “Belt Electrode-Skeletal Muscle Electrical Stimulation” (B-SES), a neuromuscular stimulation technique that stimulates large muscle areas, and focuses predominately on frail cohorts in a hospital setting^9^. While many features are comparable to WB-EMS (1), B-SES uses a monophasic, exponentially climbing pulse. Most importantly however, in contrast to WB-EMS that stimulates all main muscle groups, B-SES focuses on hip and lower extremity muscle groups, applying five [e.g., (16)] or six [e.g., (15)] belt electrodes fixed at the waist/lower back, thigh and ankles. Duration of WB-EMS and B-SES sessions were comparable, while training frequency of B-SES in the included studies is about twice as high. Stimulus intensity of B-SES was consistently described as the maximum tolerable impulse intensity without pain (or discomfort); i.e., largely in line with the specification applied by WB-EMS. For both methods, acute stimulation effects on deeper muscle layers of the thigh and lower legs were reported (46, 47). (2) As a key limitation of the systematic search, we cannot be sure that we identified all eligible articles, particularly due to poor information provided, difficulties in proper translation and, in some cases, missing author responses to our queries. This also applies for “adverse effects” that were not consistently listed by all publications or answered upon request (*n* = 4). Unfortunately, these included particularly important studies with very vulnerable cohorts (24–26, 36). (3) Due to the specific situation in Germany i.e., federal directives, mandatory trainer education and a medical WB-EMS market, we decided not to include other, non-German working groups on WB-EMS in the consensus process. In parallel, it would be inappropriate to simply transfer the present contraindications to other markets with diverging regulatory structures for non-medical WB-EMS. This particularly refers to countries with non-mandatory specific trainer qualification. (4) Similar to the 2019 list of contraindications (4), our evidence-based, consensus-generated recommendations have no mandatory character. Nevertheless, we recommend all parties to respect the present contraindications to ensure safe WB-EMS application and thus avoid an even more severe restriction (next stage) at a European Union level. (5) Even this updated list of contraindications should not be regarded as the final version. Considering the largely increased amount of WB-EMS trials with a majority of studies that focus on cohorts with limitations or diseases (1), we feel that another update of the contraindications should be performed no later than in 3–4 years.

## Footnotes

^1^Ordinance on Protection against the Harmful Effects of Non-Ionising Radiation in Human Applications (NiSV).

^2^Although the 2019 version of the contraindications (20) did not define the severity of the bleeding disorder, it is evident that only severe and life-threatening bleeding disorders should be considered as an absolute contraindication for WB-EMS. For clarity, this aspect has now been added.

^3^The reasons for focusing on the selected medical conditions are explained again in more detail in the discussion.

^4^≥50% of skeletal muscle mass.

^5^Unfortunately, no data on insulin dependent Diabetes Mellitus (DM Type I) was available.

^6^A further B-SES case control study (42) not included in the evidence map focused on the same cohort.

^7^Another case control study (43) not included in the evidence map focused on B-SES and virtual reality-guided balance training (30 days) for managing paraplegia after spinal cord infarction.

^8^According to our definition, medical WB-EMS can be considered as (1) primarily therapeutic intervention (2) based on an existing diagnosis (3) that is provided by qualified medical–therapeutic personnel (4) in compliance with current guidelines and (5) using medical device (44).

^9^According to the manufacturer (HOMERION, Japan), more than thousand hospitals and care facilities in Japan applied B-SES.
